# Vitamin D Toxicity Presenting as Altered Mental Status in Elderly Patients

**DOI:** 10.7759/cureus.32654

**Published:** 2022-12-18

**Authors:** Umar H Khan, Suhail Mantoo, Amrit Dhar, Afshan Shabir, Asma Shah, Nazia Mehfooz, Sonaullah Shah

**Affiliations:** 1 Internal and Pulmonary Medicine, Sher-i-Kashmir Institute of Medical Sciences, Srinagar, IND

**Keywords:** elderly, altered mental state, vitamin d toxicity, hypercalcemia, geriatrics

## Abstract

Background and objective

Around 25-30% of elderly patients present to emergency departments (ED) with altered mental status (AMS), with hypercalcemia being one of the metabolic causes. Elderly patients, due to their multiple vulnerability factors and relative homeostenosis, are susceptible to alterations in mental state at even milder grades of hypercalcemia. There is a trend of overzealous prescription of higher doses of vitamin D in elderly patients for various ailments, which often exceeds the requirements of the patients. In this study, we aimed to establish vitamin D toxicity (VDT) as an underlying cause of AMS in elderly patients presenting to the hospital.

Methods

This was a descriptive case study conducted at a tertiary care university hospital in North India, from January 2015 to March 2020 for a total duration of five years. Elderly patients (aged ≥60 years) who were admitted with VDT as a cause for underlying hypercalcemia were included. The evaluation included patient history regarding the dosage of vitamin D received, route of administration, and biochemical parameters, such as serum calcium, intact parathyroid hormone (iPTH), 25-hydroxy vitamin D [25(OH)D], and albumin. All other potential causes for hypercalcemia and AMS were ruled out concurrently.

Results

A total of 19 patients were enrolled in the study, with a mean age of 72.3 years (range: 62-86 years). All patients had received injectable vitamin D formulation. The mean serum calcium among the patients was 12.52 ± 1.12 mg/dL (range: 11.2-15.7 mg/dL), whereas the mean 25(OH)D was 196.34 ± 70.44 ng/mL (range: 107-356 ng/mL). The mean cumulative vitamin D supplement intake was 2.594 ± 0.841 million IU (range: 1.2 million-4.2 million IU). While six patients had mild hypercalcemia, 12 had moderate, and one person had severe hypercalcemia, with altered sensorium (85%) being the most common complaint for presenting to ED, followed by generalized weakness (15%).

Conclusion

VDT can manifest with AMS as an initial presenting complaint. The geriatric population, due to various underlying vulnerability factors, is more susceptible than their younger counterparts. We strongly recommend that in elderly patients, higher doses of vitamin D should be prescribed only after checking their serum levels, and frequent monitoring of vitamin D should be performed.

## Introduction

Altered mental status (AMS) is a common complaint among elderly patients who present to the emergency department (ED). Up to 25-30% of elderly patients present to ED with AMS, with the majority of the causes ranging from delirium to stupor to coma. Urinary tract infections and pneumonia account for the majority of known causes of acute brain dysfunction in the geriatric population. Some of the metabolic causes are hyponatremia, hypernatremia, hypoglycemia, hypercalcemia, and hyperglycemic states. Others include hepatic, uremic, and hypoxic encephalopathy, as well as thyrotoxicosis and Wernicke encephalopathy [1,2]. Hypercalcemia is reported less frequently as an initial presentation of encephalopathy, whereas hyperparathyroidism is reported in the majority of such cases [3,4]. Vitamin D toxicity (VDT) is another precipitant factor for hypercalcemia-associated encephalopathy, especially in geriatric patients; however, it is reported less frequently.

In recent years, general practitioners have been more frequently ordering tests for underlying vitamin D deficiency and prescribing significantly more oral or injectable vitamin D to treat the condition [5]. This trend of zealous prescription, particularly to the geriatric population, may result in VDT, which can manifest as AMS. The current literature on VDT is limited to case reports, case series, and animal experiments, and there is a lack of prospective studies on the condition, particularly in the geriatric subset. Our research was carried out in the Kashmir valley, where vitamin D deficiency is known to be rampant [6,7]. Its presentation varies from drowsiness to a comatose state depending on the severity and rate of hypercalcemia, and this is especially concerning in elderly patients who can develop AMS even at lower levels of serum calcium [1]. In light of this, we set out to look into VDT as a possible cause of AMS in elderly patients who presented to the hospital.

## Materials and methods

This was a single-center, descriptive case study performed between January 2015 and March 2020 at a 1200-bedded tertiary care university teaching hospital in North India. The Intradepartmental Ethics Committee at Sher-i-Kashmir Institute of Medical Sciences in Srinagar, India approved the study (approval number: SIMS-128-0132/22), and all procedures were in accordance with the tenets of the Declaration of Helsinki. The participants were enrolled after obtaining written informed consent.

Study participants

Elderly patients (aged ≥60 years) who presented with hypercalcemia to ED were screened for underlying etiology. Patients with VDT-associated hypercalcemia (serum calcium corrected for albumin of more than 10.5 mg/dl) were included. Around 385 patients presented to ED with hypercalcemia during the study period, of which 19 fit the inclusion criteria, amounting to an incidence rate of 4.9%. Other potential causes of hypercalcemia such as hyperparathyroidism, granulomatous disease, multiple myeloma, intrinsic renal disease, or malignancies were ruled out in each case based on appropriate investigations. CT (non-contrast) of the brain was performed in all patients with altered sensorium to rule out alternative acute neurological causes.

Case definitions

Hypervitaminosis D was defined as serum 25-hydroxy-vitamin D [25(OH)D] of more than 100 ng/mL and suppressed intact parathyroid hormone (iPTH) [8]. Hypercalcemia was classified into three types based on calcium levels - mild hypercalcemia: 10.5-11.9 mg/dL, moderate hypercalcemia: 12.0-13.9 mg/dL, and severe hypercalcemia: 14.0-16.0 mg/dL [9]. AMS was defined as a state of unresponsiveness, sudden behavioral change, drowsiness, or disorientation [1].

Diagnostic evaluation

Patients were managed by attending emergency physicians initially and later in the geriatric ward. The clinical evaluation with detailed history and examination was done. The history focused on the dosage, frequency, and route of administration of vitamin D. Baseline hemogram and renal function tests were done. The biochemical evaluation included the measurement of total serum calcium, iPTH, serum vitamin D, and albumin. Demographic data related to age, gender, and comorbidities were collected. Emphasis was laid on the initial presentation and associated complaints were noted. Patients were followed up till eventual discharge or mortality in the hospital. For iPTH and vitamin D status, the blood sample was collected in a clot activator vacutainer and sent immediately for analysis. Serum vitamin D was quantified by chemiluminescent immunoassay (Automated Beckman Coulter Access 2 Immunoassay System, Beckman Coulter Inc., Brea, CA). All the samples were analyzed at the same time in the same laboratory (Immunology laboratory of Sher-i-Kashmir Institute of Medical Sciences, Srinagar, India). The reference range for iPTH levels was 12-88 pg/ml.

Statistical analysis

For descriptive statistics, the continuous variable was summarized as mean and standard deviation (SD) or median and interquartile range (IQR) as appropriate. The categorical variables are presented as frequencies and percentages. All data were assessed for normality. The Kolmogorov-Smirnov test was performed to evaluate the distributions of continuous variables. Statistical analysis was done using IBM SPSS Statistics for Windows, Version 28.0 (Released 2021; IBM Corp., Armonk, NY). The significance level was set at p<0.05.

## Results

Of the total 19 patients, 10 were males and nine were females; the age of the patients ranged from 62 to 86 years (median: 71 years). Hypertension (36.8%) was the most common comorbidity. Table 1 depicts the individual parameters of all the cases.

**Table 1:** Individual baseline parameters of all patients enrolled in the study iPTH: intact parathyroid hormone; Cr: creatinine; 25(OH)D: 25 hydroxy vitamin D; AS: altered sensorium; GW: generalized weakness; P: polyuria; AN: anorexia

Variable	Case 1	Case 2	Case 3	Case 4	Case 5	Case 6	Case 7	Case 8	Case 9	Case 10	Case 11	Case 12	Case 13	Case 14	Case 15	Case 16	Case 17	Case 18	Case 19
Age in years/sex	75/F	70/M	75/F	85/F	72/M	65/M	70/F	70/F	86/F	62/M	65/M	70/M	80/F	75/M	72/M	70/F	75/M	65/F	71/M
Vitamin D dose (in million units)	3	2.4	3.6	1.2	1.8	1.8	2.4	3.0	2.4	4.2	1.8	2.4	3.0	2.4	3.6	1.8	4.2	2.4	1.8
Calcium (mg/dL)	12.3	12.5	13.5	11.3	11.3	12.6	12.6	13.9	11.4	12.1	11.2	12.5	11.5	13.5	12.8	11.3	12.3	13.7	15.7
Phosphate (mg/dL)	4.6	3.6	4.9	6.1	3.7	5.5	3.6	4.2	4.9	5.9	7.1	7.7	4.4	3.7	5.3	5.7	6.3	3.8	6.2
iPTH (pg/mL)	15	23	12.5	10	14	12.5	13.1	10	14.7	11.4	6	0.01	14	5	3.7	7.8	30	13	20.7
Urea (mg/dL)	56	86	68	45	48	78	67	24	32	36	103	48	32	64	66	33	120	33	38
Cr (mg/dL)	1.8	2.2	2.1	1.9	3.4	1.7	1.8	0.8	6.1	1.7	2.4	1.5	0.8	1.6	3.3	1.9	2.8	1.9	1.8
25(OH)D (ng/mL)	234	154	160	132	312	168	172	207	230	208	114	107	161	206	213	356	135	137	321
Presenting complaints	AS	AS	AS, P	AS	GW, P	AS, AN	AS	AS	AS	GW	AS, P	AS	AS	AS, P	AS	AS	GW	AS	AS

Table 2 depicts the general demographic and biochemical parameters of cases. The mean serum calcium among the patients was 12.52 ± 1.12 mg/dL (range: 11.2-15.7 mg/dL), with corresponding phosphates ranging between 3.6 and 7.7 mg/dL (mean: 5.11 ± 1.23 mg/dL). The mean serum vitamin D [25(OH)D] was 196.34 ± 70.44 ng/mL (range: 107-356 ng/mL). iPTH was found to be expectedly low in patients, with a mean of 12.44 ± 6.94 pg/mL (range: 0.01-30 pg/mL). Of note, 84% (n=16) of the patients had azotemia with serum creatinine ranging from 0.8 to 6.1 mg/dL (mean: 2.18 ± 1.16 mg/dL) and mean serum urea of 56.68 ± 26.18 mg/dL. Nephrolithiasis was ruled out in all patients via ultrasonography and all had normal-sized kidneys with normal echotexture and maintained corticomedullary differentiation. No evidence of chronic renal failure was seen in any of our subjects. Other causes of hypercalcemia such as hyperparathyroidism, granulomatous disease, multiple myeloma, intrinsic renal disease, or malignancies were ruled out in all cases. CT (non-contrast) of the brain was performed in all patients with altered sensorium to rule out alternative acute neurological causes, and no abnormality was detected in any of them.

**Table 2:** Biochemical parameters of the study subjects SD: standard deviation; COPD: chronic obstructive pulmonary disease; CKD: chronic kidney disease; TLC: total leucocyte count; PTH: parathyroid hormone

Variable	Value
Age in years, mean ± SD	72.3 ± 6.5
Gender	n (%)
Male	10 (52.6)
Female	9 (47.4)
Comorbidities	n (%)
Hypertension	7 (36.8)
Diabetes mellitus	4 (21.1)
COPD	5 (26.3)
Hypothyroidism	3 (15.8)
CKD	2 (10.5)
Laboratory parameters	Mean ± SD
Hemoglobin (mg/dL)	11.55 ± 2.5
TLC (cells/cubic mm)	10,190 ± 4,630
Serum creatinine (mg/dL)	2.18 ± 1.16
Sodium (mEq/L)	136.57 ± 11.10
Potassium (mEq/L)	3.34 ± 0.84
Serum calcium (mg/dL)	12.34 ± 1.19
Serum phosphorus (mg/dL)	5.11 ± 1.23
25 hydroxy vitamin D (ng/mL)	196.35 ± 70.44
Intact PTH (pg/mL)	12.44 ± 6.94
Vitamin D supplementation (in million IU)	2.594 ± 0.841

Altered sensorium was the presenting complaint to the ED in the majority of patients (85%, n=16) followed by generalized weakness in 15% (n=3). Polyuria and anorexia were seen in 22.2% and 5.6% respectively, in addition to the main presenting complaints of altered sensorium and generalized weakness. While six patients had mild hypercalcemia, 12 had moderate, and one had severe hypercalcemia. No significant difference between the severity of hypercalcemia and the initial presenting symptom was found (p>0.05), as shown in Table 3.

**Table 3:** Distribution of presenting complaint according to hypercalcemia severity No significant difference between the severity of hypercalcemia and the initial presenting symptom was found (p>0.05)

Presenting complaint	Severity of hypercalcemia		
	Mild, n (%)	Moderate, n (%)	Severe, n (%)
Altered sensorium (n=16)	5 (31)	10 (62)	1 (6)
Generalized weakness (n=3)	1 (33)	2 (67)	0
Polyuria (n=4)	2 (50)	2 (50)	0
Anorexia (n=1)	0 (0)	1 (100)	0

All patients had received multiple intramuscular injections of vitamin D, each containing a dose of 600,000 IU. The mean cumulative vitamin D supplement intake was 2.594 ± 0.841 million IU (range: 1.2 million-4.2 million IU). Of note, 17 patients had received supplementation on the advice of general practitioners while two had self-medicated on the advice of local pharmacists. None of the patients had a biochemical assessment of vitamin D done before supplementation.

In our study, all patients received hydration and glucocorticoids, whereas eight patients were given calcitonin. Three of the patients eventually succumbed to sepsis and multi-organ failure; follow-up was traceable for only three of the remaining patients, all of whom still had vitamin D levels of more than 100 ng/mL at six months. During the next follow-up at 12 months, one patient had vitamin D of less than 100 ng/mL, while the other two patients still had elevated serum levels.

## Discussion

Vitamin D is a pro-hormone, which plays a crucial role in calcium homeostasis and bone metabolism, and is important for cell differentiation and immune modulation [10]. The daily estimated average requirement (EAR) and recommended dietary allowance (RDA) for vitamin D are around 400 IU (10 μg) and 600 IU (15 μg) respectively for adults; however, for people aged more than 70 years, RDA is higher, i.e, 800 IU (20 μg). The Food and Nutritional Board of the USA has set the current tolerable upper limit of intake at 4000 IU/day (100 μg) [11]; however, several studies have suggested that intake to the level of 10,000 IU/day for prolonged periods could be safe. Many reports have elucidated the VDT threshold to be between 10,000 and 40,000 IU/day and serum levels greater than 150 ng/mL (>375 nmol/L); however, due to this wide therapeutic index, toxicity is rarely reported [11-13]. In our study, the mean vitamin D level observed was around 196.35 ng/mL, which was well above the current established toxicity level. The mean cumulative dose of vitamin D was 2.59 million IU, which is more than the recommended dose for vitamin D deficiency.

Common presentations of VDT are the same as those of hypercalcemia, i.e., polyuria, increased thirst, fatigue, muscle cramps, myalgia, nausea, vomiting, bony pains, constipation, and AMS. However, there is considerable variability in how patients present, depending on the magnitude and rate of calcium increase [9]. In our study, the most common presentation at ED was AMS (delirium, agitation, psychosis, stupor, coma), seen in 85% of total subjects, followed by generalized weakness in 15%. Other accompanying manifestations included polyuria, anorexia, and vomiting. In a similar study by Misgar et al., neurological manifestations in VDT were seen in only 37% of cases, whereas gastrointestinal manifestations were the most common (93%) [14]. In our study, almost all patients with mild and moderate hypercalcemia presented with AMS, which is generally associated with severe hypercalcemia. This difference can be attributed to the fact that the mean age in our study was higher, as our study focused on VDT in the geriatric population. Elderly patients are more likely to have multiple underlying vulnerability factors and are therefore more susceptible to alteration in mental status due to their relative homeostenosis. The vulnerability factors include underlying dementia, advanced age, poor functional status, malnutrition, high comorbidity index, and the use of psychoactive medications [2,15].

Alteration in mental status in the form of cognitive dysfunction is often observed in moderate hypercalcemia, whereas severe hypercalcemia is known to manifest as lethargy, confusion, stupor, delirium, and coma. The underlying mechanism is not clear, but it is believed that hypercalcemia induces a state of excitotoxicity, acting as a catalyst for neuronal demise and altering the levels of various neurotransmitters. This excitotoxicity mediated via glutaminergic neurons through N-methyl-D-aspartate (NMDA) receptors is another possible explanation for hypercalcemia-induced AMS and psychosis. NMDA receptor-mediated influx of calcium thus contributes to neuronal demise by facilitating increased mitochondrial permeability, leading to mitochondrial rupture, cell dysfunction, and death. In the majority of cases, it has been found that restoring normocalcemia rapidly resolves neuropsychiatric symptoms [16,17].

Researchers have come up with multiple hypotheses regarding the mechanism behind VDT, all of which involve increased concentrations of vitamin D metabolite reaching the vitamin D receptor in the nucleus of target cells and subsequently leading to an exaggerated gene expression. Plasma 1-alpha-25(OH)D concentrations get elevated on vitamin D intake, which in turn increases cellular 1-alpha-25(OH)D concentrations, as described by Jones [13]. Also, vitamin D intake raises plasma 25(OH)D to concentrations that exceed the binding capacity of vitamin D binding protein and, subsequently, “free 25(OH)D” enters the cell, having direct effects on gene expression.

VDT is a medical emergency, especially in geriatric patients who present with AMS and hypercalcemia, and VDT should be thought of as one of the potential etiologies. Intravenous hydration with normal saline constitutes the mainstay of treatment and glucocorticoids are used as well. Additionally, bisphosphonates and calcitonin have been used to treat VDT [9]. Hypercalcemia due to VDT takes a long time to normalize as vitamin D has a slow release from fat deposits. The average whole-body half-life of vitamin D is 62 days [18]. In one study, the resolution of hypercalcemia took a median of seven months (range: 4-18 months) [14]. Other case reports and series have been published on VDT, but none of them has looked into the time for resolution of hypercalcemia on follow-up.

The incidence of VDT is on the rise and it is now recognized as a not-so-uncommon cause of hypercalcemia. There are multiple reasons for this; firstly, the growing awareness among the common masses presumably regarding skeletal and non-skeletal benefits of vitamin D leads to a propensity for overprescription for elderly patients who often present with non-specific aches. In our study, almost all subjects were overzealously prescribed multiple injections of vitamin D for non-specific body aches and fatigue, while none of the patients had received oral vitamin D. Second, the lack of understanding of pharmacokinetics and pharmacodynamics of vitamin D in the elderly population leads to frequent overdoses. Third, the easy availability of over-the-counter formulations makes things worse.

Our study was limited by an observational design, a relatively small size, and it being a single-center study. Also, ours is a tertiary care hospital, and hence many cases might not consult us and would be seen or managed at the peripheral level; hence, the incidence of VDT in our study may not be representative of the wider populace. However, ours is the first study looking into VDT and its presenting complaints in the elderly population from our region.

## Conclusions

VDT can manifest with AMS as an initial presenting complaint. The geriatric population, due to various underlying vulnerability factors, is more susceptible than their younger counterparts to the effects of hypercalcemia, which can even manifest at comparatively lower levels of serum calcium. We strongly recommend that higher doses of vitamin D should be prescribed only after checking serum levels and frequent monitoring of vitamin D should be done in elderly patients.
